# Megadose Methylprednisolone (MDMP) For Granulocytic Sarcoma (GS)

**DOI:** 10.4274/tjh.03779

**Published:** 2013-03-05

**Authors:** Şinasi ÖZSOYLU

**Affiliations:** 1 Retired Professor of Pediatrics, Hematology, and Hepatology; 2 Honorary Fellow of American Academy of Pediatrics; 3 Honorary Member of American Pediatric Society; 4 Fellow of Islamic World Academy of Sciences

**To the Editor,**

Baytan et al.’s case report entitled, Cerebellar granulocytic sarcoma: A case report (2012; 19: 177-180), [1] provides an opportunity for me to remind physicians that short-course megadose methylprednisolone (MDMP: 30 mg/kg for 3 d, then 20 mg/kg for 4 d each dose administered over the course of 10-15 min intravenously or orally; calculated dose of powder put into a spoon and then covered with honey and administered before 0600), has been effectively used for the treatment of granulocytic sarcoma (GS) [2,3,4,5]. With this treatment extramedullary relapse rarely occurs, even in patients that develop bone marrow relapse [2].

Administration of MP early in the morning usually prevents the occurrence of steroidal side effects, most likely due to preservation of ACTH steroid homeostasis. This treatment should be considered for the treatment of GS, as it is less expensive, more convenient, and easy to administer orally at home. I would like to emphasize that to the best of my knowledge neutrophilic sepsis has not been reported in association with this treatment [2,5]. 
